# Hsp90β knockdown in DIO mice reverses insulin resistance and improves glucose tolerance

**DOI:** 10.1186/s12986-018-0242-6

**Published:** 2018-02-02

**Authors:** Enxuan Jing, Pragalath Sundararajan, Ishita Deb Majumdar, Suwagmani Hazarika, Samantha Fowler, Angela Szeto, Stephane Gesta, Armando J. Mendez, Vivek K. Vishnudas, Rangaprasad Sarangarajan, Niven R. Narain

**Affiliations:** 1 BERG, LLC, 500 Old Connecticut Path, Bldg B (3rd Floor), Framingham, MA 01701 USA; 2 Diabetes Research Institute, University of Miami Miller School of Medicine, University of Miami, Coral Gables, FL USA; 3 Diabetes Research Institute and Division of Endocrinology, Diabetes, and Metabolism, University of Miami Miller School of Medicine, 500 Old Connecticut Path, Bldg B (3rd Floor), Miami, FL 33136 USA

**Keywords:** Heat shock protein 90, Diabetes, Hyperglycemia, Insulin resistance, Glucose metabolism

## Abstract

**Background:**

Inhibition of Hsp90 has been shown to improve glucose tolerance and insulin sensitivity in mouse models of diabetes. In the present report, the specific isoform Hsp90ab1, was identified as playing a major role in regulating insulin signaling and glucose metabolism.

**Methods:**

In a diet-induced obese (DIO) mouse model of diabetes, expression of various Hsp90 isoforms in skeletal tissue was examined. Subsequent experiments characterized the role of Hsp90ab1 isoform in glucose metabolism and insulin signaling in primary human skeletal muscle myoblasts (HSMM) and a DIO mouse model.

**Results:**

In DIO mice *Hsp90ab1* mRNA was upregulated in skeletal muscle compared to lean mice and knockdown using anti-sense oligonucleotide (ASO) resulted in reduced expression in skeletal muscle that was associated with improved glucose tolerance, reduced fed glucose and fed insulin levels compared to DIO mice that were treated with a negative control oligonucleotide. In addition, knockdown of HSP90ab1 in DIO mice was associated with reduced pyruvate dehydrogenase kinase-4 mRNA and phosphorylation of the muscle pyruvate dehydrogenase complex (at serine 232, 293 and 300), but increased phosphofructokinase 1, glycogen synthase 1 and long-chain specific acyl-CoA dehydrogenase mRNA. In HSMM, siRNA knockdown of Hsp90ab1 induced an increase in substrate metabolism, mitochondrial respiration capacity, and insulin sensitivity, providing further evidence for the role of Hsp90ab1 in metabolism.

**Conclusions:**

The data support a novel role for Hsp90ab1 in arbitrating skeletal muscle plasticity via modulation of substrate utilization including glucose and fatty acids in normal and disease conditions. Hsp90ab1 represents a novel target for potential treatment of metabolic disease including diabetes.

**Electronic supplementary material:**

The online version of this article (10.1186/s12986-018-0242-6) contains supplementary material, which is available to authorized users.

## Background

The heat shock proteins (HSPs) constitute a group of stress proteins typically induced to counteract the deleterious effects of heat on intracellular structure and function [1, 2]. Interestingly, heat shock response can be activated by a temperature increase of a just a few degrees; a generally rapid and transient gene-expression response and the kinetics of this response might vary depending on the HSPs involved [3]. However, it is now recognized that HSPs respond to a multitude of stress signals ranging from that induced by chemical, environmental, and physical to that of physiological origin. Their chaperone function linked to protection of protein structure and function [1, 4] positions HSPs as a central participant in nearly all physiological processes ranging from cell cycle to intracellular signaling and regulation of metabolism.

Prior studies implicate that Hsp90 plays a critical role in metabolism. Specifically, treatment with a pan-Hsp90 inhibitor was shown to improve glucose homeostasis and insulin sensitivity in db/db and diet-induced obese (DIO) mouse models of diabetes, respectively [5]. However, the Hsp90 family consists of multiple isoforms including Hsp90aa1, Hsp90ab1, Hsp90B1, and TRAP1 that are ATP dependent chaperone proteins [6–8]. The more extensively studied Hsp90 isoform, Hsp90aa1, is a major cancer target that has been shown to be upregulated and involved in chaperoning multiple proteins including important proliferative signaling and mitochondrial membrane proteins [9, 10]. In contrast, less is known about Hsp90ab1, but it has been reported to be constitutively expressed at low levels [9, 11]. Thus, given that isoform specific differences in expression and function exist [9, 10], the present study sought to determine if the regulation of Hsp90 on metabolism is isoform specific. The findings presented in this study show that the HSP90ab1 isoform is upregulated in skeletal muscle of DIO mice. Furthermore, isoform specific knockdown of HSP90ab1 by treatment with antisense oligonucleotide (ASO) improved glucose tolerance and was associated with alterations in expression and activity of key metabolic pathway molecules in skeletal muscle of DIO mice. Furthermore, in in vitro studies using primary human skeletal muscle myotubes (HSMM) siRNA knockdown of HSP90ab1 was associated with significant improvement in substrate metabolism, insulin sensitivity and glucose homeostasis. In addition, knockdown of Hsp90ab1 does not affect the expression of proteins within the canonical Hsp90 pathway that includes HSF-1, Hsp47, TRAF1 and Hsp70. Together, these data demonstrate that Hsp90ab1 is a key regulator of skeletal muscle metabolism and represents a viable therapeutic target for management of insulin resistance and metabolic disease.

## Methods

### Animals

Male C57BL/6 J mice were obtained from Jackson Laboratories (Bar Harbor, ME) and housed 4-5 per cage at 22 °C on a 12:12 h day-night cycle. At 6 weeks of age mice were fed a high-fat diet (Research Diets Cat #: D12492; 60% kcal fat, 20% kcal protein, and 20% kcal carbohydrate). At 8 weeks of age animals received and continued a high fat diet during the entire study period. Lean control mice were also obtained and fed a standard diet (10% kcal fat). Mice were acclimated to the local facility for 1 week before treatment. Antisense oligonucleotides (ASOs) were administered at 10 μg/kg intraperitoneally (i.p.) twice a week for 4 weeks. The procedures for the care and use of experimental animals followed the protocols and regulations set forth by the Animal Care and Use Committee of the University of Miami.

### Cell culture

Primary human skeletal muscle myoblasts (HSMM) were purchased from Promocell and maintained in ready to use Promocell Growth Media (Promocell, c-23,060) at 37 °C in a 5% CO_2_ incubator. Myoblasts were differentiated on petri dishes with differentiation media containing 2% horse serum (Invitrogen, Carlsbad, CA) and grown for 6 days before experiments. Differentiated HSMM were transiently transfected with scrambled control and siRNA targeting human Hsp90ab1 for 48 h using Transit TKO siRNA transfection reagent (Mirus Bio, Madison, WI) according to manufacturer’s recommendations. Knockdown was confirmed using quantitative real time PCR and Western blot.

### Quantitative real time polymerase chain reaction (qRT-PCR)

RNA was extracted using RNeasy (QIAGEN). The cDNA was synthesized using 1 μg total RNA using the All Advantage RT-PCR kit. Five microliters of cDNA was used for quantitative PCR using Sybrgreen master mix (Applied Biosystems) on a Biorad thermal cycler. DeltaCt (dCt) values determined after normalization against either 18S ribosomal RNA or cyclophilin A. The dCt values were calculated using absolute Ct values of the normalizer subtracted by Ct values of target genes. Final values were calculated using 2 exponential to the −dCt. Each condition was performed in triplicate.

### SDS-PAGE and western blot

Cell lysates were fractionated by 10% SDS-PAGE then transferred to PVDF membranes (Invitrogen). After blocking for 1 h at room temperature, the membranes were incubated overnight at 4 °C in primary antibody. The membranes were incubated with 1:2000–1:10,000 secondary antibodies conjugated with HRP for 1 h at room temperature after washing 3 times for 10 min each. Signals were detected using the Pierce ECL (ThermoFisher) chemiluminescence system and visualized by autoradiography.

### Enzyme-linked Immunosorbent assay (ELISA)

Following knockdown of Hsp90ab1, cells were serum-starved for 3 h in serum-free basal media (Promocell, Germany) containing 0.2% BSA prior to stimulation with insulin for 5 min, washed with cold PBS, then lysed with cell lysis buffer provided in the Instant One ELISA kit (eBioscience). Protein concentration was determined using a BCA assay (Thermo Fisher). ELISA for p-Akt and Akt was performed using Instant One ELISA kit. Results are expressed as p-Akt:Akt ratio for each condition.

### Fatty acid oxidation (FAO) assay, bioenergetics profiling and glycostress assays

Cellular oxygen consumption rate (OCR) was measured using a Seahorse Bioscience XF96 flux analyzer. Myoblasts seeded at 10,000 cells/well were grown up to 80% confluence before being differentiated with 2% Horse media for 4 days. The cells were then transfected with either scrambled control or Hsp90AB1 siRNA in differentiation media for 48 h prior to analysis. Replicates (3-6) were performed for each experimental condition. For OCR experiments that used palmitate, KHB buffer containing L-carnitine (final concentration 500 μmol/L) (pH 7.4) was added to each well and measurements were performed every 3 min with 2 min inter-measurement mixing. BSA-conjugated palmitate (final concentration 400 μmol/L), CCCP (final concentration 1 μmol/L) and etomoxir (final concentration 50 μmol/L) were injected sequentially. For glycostress experiments, glucose was used as a substrate with sodium carbonate and glucose/pyruvate-free DMEM. Glucose, Oligomycin and 2-DG were injected sequentially at final concentrations of (25 mmol/L, 2 μmol/L and 50 mmol/L respectively) extracellular acidification rate (ECAR) was recorded.

### Intraperitoneal glucose tolerance test (IPGTT)

Glucose tolerance test was performed after a 6 h fast in the morning. Initial fasting blood glucose levels were determined followed by intraperitoneal (i.p.) injection of 20% dextrose solution (1.5 g/kg body weight). Blood glucose levels were measured from the tail vein at 15, 30, 60, 90, and 120 min after glucose injection. During the IPGTT experiments, different groups were performed in parallel in the same assay.

### Measurements of glucose, insulin and glycogen

Blood glucose levels were measured using an Accu-chek Advantage glucometer (Roche Diagnostics, Indianapolis, IN). Insulin was measured weekly using serum obtained from the tail vein using an insulin ELISA assay kit (Rat/mouse insulin assay kit, Mercordia, Winston-Salem, NC). Body weight and fed blood glucose levels were measured twice weekly, and fed insulin was measured weekly. Glycogen levels were assessed using an ELISA kit (BioVision, San Francisco, CA).

### Data analysis

GraphPad Prism was used for calculations of area under the curve (AUC) and for statistical analysis. For comparisons between two groups Student’s t-test were performed. For time-course IPGTT studies repeated measures ANOVA were performed. A *p*-value < 0.05 was deemed significant.

## Results

### Diet-induced obesity induces Hsp isoform specific alterations in skeletal muscle

**Fig. 1:**
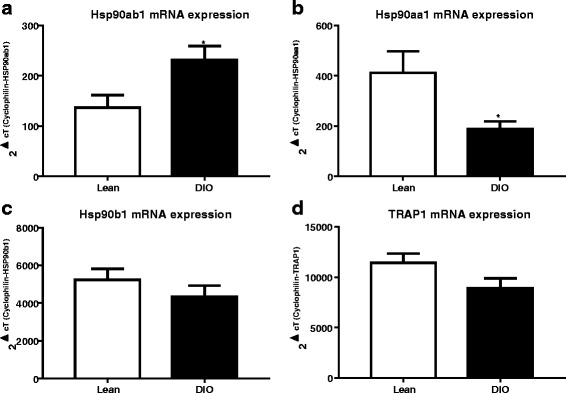
Isoform specific alterations in Hsp90 mRNA after diet-induced obesity (DIO). Male C57B/6 mice at 8 week old were fed with either a 10% kcal fat (Lean) or a 60% kcal fat high fat diet (DIO) for 12 weeks. After an 18 h fast mice were sacrificed and skeletal muscle were analyzed by quantitative real time PCR (qPCR) analysis for (**a**) *Hsp90ab1*, (**b**) *Hsp90aa1*, (**c**) *Hsp90b1*, and (b) *TRAP1* mRNA. Data represents mean + standard error of the mean (SEM) of *n* = 5 (Lean) and *n* = 6 (DIO) mice. *p < 0.05, Student’s t-test

The expression of various isoforms of Hsp90 was examined to determine if Hsp regulation of metabolism is isoform specific. In these studies mRNA expression of *Hsp90ab1*, *Hsp90aa1*, *Hsp90b1*, and *TRAP1* were assessed from skeletal muscles of mice fed a high fat diet (diet-induced obesity, DIO) and compared to lean counterparts. In DIO mice *Hsp90ab1* was significantly upregulated, while *Hsp90aa1* was downregulated (Fig. 1a–p< 0.05). There were no significant changes in *Hsp90b1* and *TRAP1* mRNA expression in DIO compared to lean mice (Fig. 1c and d). *Hsp90aa1* (HSP90α) is known to be inducible, while *HSP90ab1* (HSP90β) is traditionally thought to be constitutive [8, 12]. Therefore, it is of interest to further examine whether the nutrition-mediated alteration in HSP90β could be uniquely involved in skeletal muscle metabolism.

### HSP90ab1 isoform influences glycolytic and mitochondrial metabolism in vitro

**Fig. 2:**
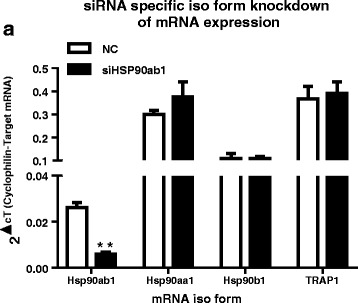
siRNA mediated knockdown of Hsp90ab1 is isoform specific in human skeletal muscle myotubes (HSMM). HSMM were transiently transfected with siRNA targeting Hsp90ab1 (siHsp90ab1) or negative control (NC) siRNA for 48 h prior to qPCR analysis of *Hsp90ab1*, *Hsp90aa1*, *Hsp90b1*, *TRAP1* mRNA. siRNA knockdown of Hsp90ab1 was isoform specific as mRNA expression of other Hsp90 isoforms were not affected. Data represent mean + SEM of *n* = 3 independent experiments. **p < 0.01 vs. NC, Student’s t-test

**Fig. 3:**
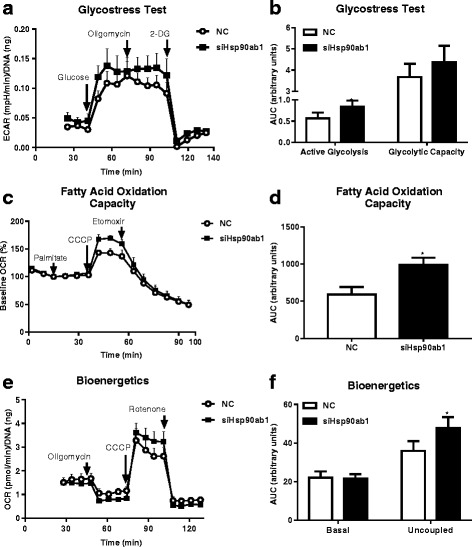
Hsp90ab1 siRNA knockdown in HSMM increases substrate metabolism and mitochondrial respiration. **a** Seahorse extracellular acidification rate (ECAR) curve from a glycolysis stress test of HSMM transfected with either negative control (NC) siRNA or siRNA to Hsp90ab1 (siHsp90ab1) measured over time. The ECAR curve shows the active glycolytic activity and maximal capacity of glycolysis obtained by sequential injections of glucose (25 mM) and oligomycin (2 μM), respectively. Injection of 2-DG (50 mM) reversed the glucose-induced ECAR increase. Data represent mean + SEM of *n* = 3 independent samples. *p < 0.05 and **p < 0.01 vs. NC, Student’s t-test. **b** Area under the curve (AUC) of ECAR/DNA glycolysis stress test after glucose and oligomycin injections after adjustment from baseline AUC (before glucose injection) plotted as fold change relative to results of HSMM transiently transfected with NC siRNA. HSMM transfected with siHsp90ab1 show increased glucose- and oligomycin-induced glycolysis. **c** Oxygen consumption rate (OCR) curve from a Seahorse fatty acid oxidation (FAO) assay measured over time. Palmitate (400μM), CCCP (1μM), and etomoxir (50μM) were sequentially injected into the assay plate containing HSMM transiently transfected with NC or siHsp90ab1. Data represent *n* = 6 independent samples. **d** Uncoupled mitochondrial respiration measured by AUC of OCR/protein after CCCP injection adjusted by subtracting basal OCR AUCs (prior to palmitate injection) plotted as fold change relative to results of HSMM transiently transfected with NC siRNA. HSMM transfected with siHsp90ab1 show increased uncoupling relative to NC. Data represent mean + SEM of n = 6 independent samples. *p < 0.05 vs NC, Student’s t-test. **e** OCR curve for bioenergetics profiling of HSMM. Oligomycin (2μM), CCCP (1μM), and rotenone (2μM) were sequentially injected and OCR was measured over time. **f** Basal and uncoupled mitochondrial respiration. AUC of OCR/DNA was calculated by subtracting AUC after rotenone injections (which represents non-mitochondrial respiration) from AUC prior to oligomycin injection (basal mitochondrial respiration, left side of graph) or AUC after CCCP injection that was adjusted after subtracting AUC prior to oligomycin (uncoupled mitochondrial respiration, right side of graph) and plotted as fold change relative to results of HSMM transiently transfected with NC siRNA. HSMM transfected with siHsp90ab1 show no difference in basal mitochondrial respiration compared to NC. In contrast, siHSP90ab1 knockdown increased uncoupled mitochondrial respiration. Data represent mean + SEM of n = 3 independent samples. *p < 0.05 vs. NC, Student’s t-test

Next, knockdown of Hsp90ab1 expression in HSMM by siRNA was used to establish a working model system to validate its role in metabolic regulation (Fig. 2). Treatment of HSMM with siRNA specific for the Hsp90ab1 isoform (siHsp90ab1) resulted in ~ 80% knockdown of *Hsp90ab1* mRNA without affecting other Hsp90 isoforms including *Hsp90aa1*, *Hsp90B1*, and *TRAP1* compared to treatment with a negative control (NC) siRNA (*p* < 0.01). Hsp90ab1 siRNA knockdown was then utilized to assess the effect of Hsp90ab1 on cellular substrate metabolism, with metabolic profiling assays (glycostress assay, palmitate oxidation assay and bioenergetics profiling assays). In the glycostress test, compared to NC, Hsp90ab1 knockdown was associated with an increase in the extracellular acidification rate (ECAR) after the addition of glucose (~ 26% increase, *p* = 0.004, Fig. 3a), indicating an increase in glucose stimulated glycolysis (Fig. 3a and b). The addition of glucose is reflective of the glucose driven glycolytic activity essential to meet the energy needs of the cells. This increase in glycolysis was minimally affected by the presence of oligomycin, an inhibitor of mitochondrial ATP synthesis (Fig. 3a and b), indicative of similar glycolytic capacities between the cell lines.

Oxygen consumption rate (OCR) in the presence of palmitate (400μM) and the mitochondrial uncoupler CCCP (1μM) was used to determine fatty acid oxidation (FAO) capacity. Hsp90ab1 knockdown induced an increase in OCR after successive injection of palmitate and CCCP compared to control (Fig. 3c and d, 11% increase, *p* = 0.025). The increased uncoupled OCR in the presence of palmitate as an oxidation substrate, suggests that Hsp90ab1 knockdown enhances β-oxidation capacity when mitochondrial respiration reaches maximal capacity. Because glycolysis and lipid oxidation supply essential energetic substrates to mitochondrial oxidative phosphorylation for ATP production, electron transport chain activity was examined to determine mitochondrial oxidative capacity in a non-substrate limiting condition (in the presence of glucose, pyruvate and glutamine). As shown in Fig. 3e and f, there was no significant difference in mitochondrial respiration between Hsp90ab1 knockdown and control in the basal state. Although addition of oligomycin did not induce differences in OCR between siHsp90ab1 and control (Fig. 3e), injection of CCCP resulted in a significant increase in OCR in cells with HSP90ab1 knockdown (Fig. 3e, and f, *p* < 0.05). This suggests that HSP90ab1 knockdown in skeletal muscle is associated with increase in the maximal respiration capacity in skeletal muscle cells.

**Fig. 4:**
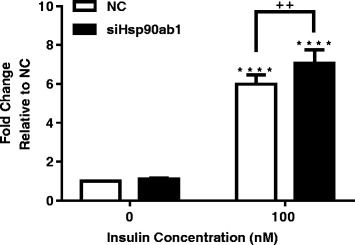
Hsp90ab1 siRNA knockdown in HSMM increases insulin signaling. HSMM were transiently transfected with either NC siRNA or Hsp90ab1 siRNA for 48 h, serum starved for 3 h, and stimulated with 0 or 100 nM insulin for 5 min. Phosphorylated-Akt was measured by ELISA, normalized by total protein, and plotted as fold change relative to NC. HSMM transfected with siHsp90ab1 show increased insulin-induced phosphorylation of Akt relative to NC. Data represent mean + SEM of n = 6 independent samples. ****p < 0.0001 vs. 0 of respective cell line. ++*p* < 0.01 vs NC

Along with substrate metabolism, insulin signaling plays a role in regulating skeletal muscle metabolic activity. Insulin stimulated Akt phosphorylation was greater with siHsp90ab1 compared to control (Fig. 4, *p* < 0.0001). Together, these results indicate that knockdown of Hsp90ab1 in HSMM increases substrate utilization capacity from glycolytic and fatty acid oxidation pathways.

### Antisense oligonucleotide (ASO) mediated Hsp90ab1 knockdown is associated with improved glucose tolerance in DIO mice

**Fig. 5:**
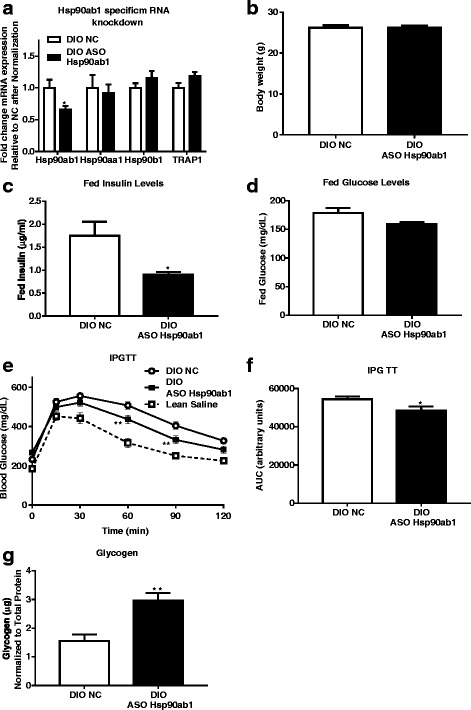
Anti-sense oligonucleotide (ASO) mediated Hsp90ab1 knockdown improves glucose tolerance and lowers levels of fed insulin and glucose in DIO mice. **a** Male mice were fed a high fat diet (DIO) for 12 weeks prior to receiving either negative control (NC) ASO or Hsp90ab1 ASO 10μg/kg/day two times a week for 4 weeks. Mice were then sacrificed, skeletal muscles were collected, and quantitative real time PCR was performed for mRNA expression of *Hsp90ab1*, *Hsp90aa1, Hsp90b1 and TRAP1* and normalized to 18 s. ASO treatment decreases Hsp90ab1 mRNA expression (*p < 0.05). There is no effect upon the mRNA expression of Hsp90aa1, Hsp90b1 and TRAP1. Data represent mean + SEM of *n* = 10/group. **b** Body weight of DIO mice treated with NC or Hsp90ab1 ASO. **c** Bar graph of fed insulin levels after 4 weeks of ASO treatment in DIO mice shows a decrease in Hsp90ab1 treated mice. **d** Bar graph of fed glucose levels after 4 weeks of ASO treatment in DIO mice. DIO mice treated with Hsp90ab1 ASO show significant improvement in glucose tolerance. **e** Blood glucose levels measured over time during an intraperitoneal glucose tolerance test (IPGTT) in lean control mice and DIO mice administered either NC ASO (n = 10) or Hsp90ab1 ASO (n = 10) after fasting for 6 h. Blood glucose was measured periodically over a time course of 120 min. Hsp90ab1 knockdown decreased blood glucose at 60 and 90 min over untreated DIO mice (**p < 0.01, *n* = 5). **f** Area under the curve (AUC) analysis of IPGTT results from data in (**e**) shows a significant overall decrease in blood glucose in Hsp90ab1-knockdown mice (*p < 0.05). **g** Glycogen levels from skeletal muscles (gastrocnemius) of DIO mice after 4 weeks of ASO treatment. DIO mice treated with Hsp90ab1 ASO show increased glycogen levels compared to mice treated with NC (**p < 0.01, *n* = 8 per group). (**g**)

An HSP90ab1 specific ASO was used to demonstrate proof-of-principle of the potential therapeutic efficacy of HSP90ab1 knockdown in improving insulin sensitivity and glucose homeostasis in DIO mice. Hsp90ab1 ASO induced a decrease in *Hsp90ab1* mRNA expression in skeletal muscle by ~ 35% (Fig. 5a, *p* = 0.02), without affecting mRNA expression of heat shock factor (HSF)-1 target genes *Hspaa1, Hsp90b1* and *TRAP1* (Fig. 5a). In addition, there were no changes in body weight between mice treated with Hsp90ab1 ASO and NC (Fig. 5b). Hsp90ab1 ASO was associated with decreases in fed insulin (Figure 5c) and a trending decrease in fed glucose levels (10% decrease, *p* = 0.056, Fig. 5d). The ASO-induced decrease in Hsp90ab1 was associated with improvement in glucose tolerance compared to control ASO (Fig. 5e and f, *p* = 0.04). Notably, significant differences between NC and ASO was observed at 60 min and 90 min during the IPGTT, reflective of differences in glucose clearance between treatment groups (Fig. 5e). Interestingly, glycogen content was significantly increased in Hsp90ab1 ASO treated DIO mice (Fig. 5g, *p* < 0.01). Thus, these data suggest that knockdown of HSP90ab1 in skeletal muscle in DIO mice is associated with improved glucose homeostasis along with overall improvement in key indices underlying onset of insulin resistance and diabetes.

### ASO mediated Hsp90ab1 knockdown in DIO mice is associated with altered expression of key genes regulating metabolic substrate utilization

**Fig. 6:**
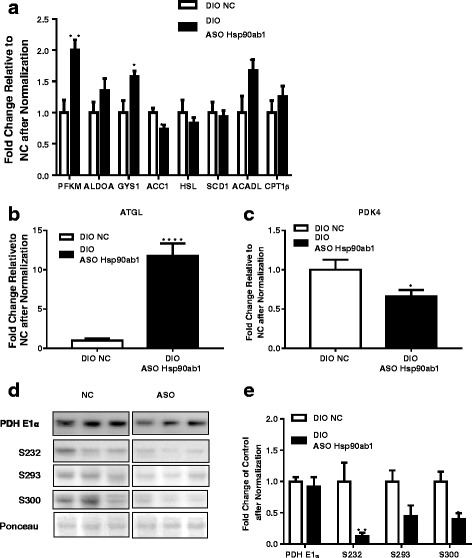
Hsp90ab1 knockdown alters expression of metabolic genes and decreases PDH catalytic subunit phosphorylation. **a** Quantitative PCR analysis of mRNA expression of metabolic enzymes for glucose and lipid metabolism in skeletal muscle of DIO mice after 4 weeks antisense oligonucleotide (ASO) treatment at 10μg/kg/day shows that PFKM, GYS1 and ACADL are significantly increased over control (*p < 0.05, **p < 0.01, *n* = 10 for NC and *n* = 9 for ASO). **b** Quantitative PCR analysis of adipose triglyceride lipase (*ATGL*) mRNA expression shows a significant increase in response to Hsp90ab1 knockdown (*n* = 10 for NC and *n* = 9 for ASO, ****p < 0.0001). **c** Quantitative PCR analysis of *PDK4* mRNA expression shows a significant decrease in response to Hsp90ab1 knockdown (*p < 0.05, *n* = 10 for NC and *n* = 9 for ASO). **d** Representative Western blot analysis of total and phosphorylated PDH E1α, the catalytic subunit of PDH complex, in muscles of DIO mice treated with NC ASO or Hsp90ab1 ASO (*n* = 5 per group). Phosphorylation of PDH E1α was examined at serine 232 (S232), serine 293 (S293), and serine 300 (S300). **e** Quantification of immunoreactive bands relative to total protein loaded as indicated by Ponceau S stain shows S232 and S300 are reduced in response to Hsp90ab1 over control (*p < 0.05, **p < 0.01, **** *p* < 0.0001 vs. NC, *n* = 5)

Next, the specific metabolic pathway(s) affected by knockdown of Hsp90ab1 in skeletal muscle was investigated. HSP90ab1 ASO treatment was associated with a significant increase in mRNA expression of skeletal muscle specific forms of phosphofructokinase (*PFKM*, ~ 100%; *p* = 0.002), glycogen synthase 1 (*GYS1*, ~ 60%; *p* = 0.013) and acyl-CoA dehydrogenase, long chain (*ACADL*, ~ 70%; *p* = 0.045) (Fig. 6a) compared to control ASO. Furthermore, mRNA expression of acetyl-CoA carboxylase alpha (*ACCA*) was decreased (Fig. 6a, *p* = 0.018), while expression of lipase hormone-sensitive (*HSL*) and carnitine palmitoyltransferase 1B (*CPT1B*) were not affected. Notably, a 10-fold increase in patatin-like phospholipase domain containing 2 (*PNPLA2*, also known as adipocyte triglyceride lipase: *ATGL*) (Fig. 6b) was observed in the Hsp90ab1 ASO treated group compared to control (*p* < 0.0001). In contrast, HSP90ab1 ASO was associated with ~ 40% decrease in expression of pyruvate dehydrogenase kinase isozymes (*PDK4*) (Fig. 6c, *p* < 0.05). Together, the alterations in expression of genes regulating glycolysis and lipid oxidation suggest that HSP90ab1 knockdown in DIO mice results in metabolic rewiring that appears to influence glucose and lipid homeostasis pathways.

Given the established role of PDK4 in influencing pyruvate dehydrogenase (PDH) activity [13], phosphorylation of the PDH catalytic subunit PDH E1α, a substrate of PDK4 was investigated. In the skeletal muscle of DIO mice treated with Hsp90ab1 ASO levels of phosphorylated PDH E1α at serine residues S232, S293 and S300 were reduced by ~ 80%, *p* = 0.021; ~ 55%, *p* = 0.055; ~ 50%, *p* = 0.011, respectively (Fig. 6d and e). More importantly, these changes were independent of total PDH E1α protein, demonstrating a bona fide regulation of PDH E1α phosphorylation and activity as a consequence of decreased PDK4 expression with Hsp90ab1 knockdown (Fig. 6d and e). These results suggest that the enhanced pyruvate dehydrogenase complex activity is associated with increased glucose oxidation in skeletal muscles, which may potentially be contributing to improved glucose homeostasis. Furthermore, recapitulation of the effects of siHsp90ab1 on PDH E1α phosphorylation status in HSMM confirms the underlying cell autonomous effects of Hsp90ab1 knockdown in orchestrating substrate utilization in skeletal muscle (Additional file 1: Figure S1). Taken together, the data demonstrates that knock-down of HSP90ab1 in skeletal muscle of DIO mice is associated with changes in gene expression patterns with ability to influence substrate utilization.

## Discussion

Type 2 diabetes mellitus is a multifactorial disease caused by genetic and environmental factors that is characterized by insulin resistance and hyperglycemia [14]. Inhibition of Hsp90 has been shown to improve insulin resistance in mouse models of diabetes [5]. In this study, the data presented here demonstrate that Hsp90ab1 is a major isoform that plays a role in regulating metabolism. Most notably, in these studies knockdown of Hsp90ab1 by ASO improved glucose tolerance, altered expression of key metabolic genes, and enhanced pyruvate dehydrogenase complex activity in a DIO mouse model. Thus, HSP90ab1 represents an actionable target that could translate into potential therapeutic benefit for diabetes.

Hsp90ab1 belongs to the family of Hsp90 proteins including Hsp90aa1, Hsp90ab1, Hsp90B1, and TRAP-1. Hsp90 proteins have been the focus of active research for multiple diseases and studies indicate that Hsp90 plays a role in biological and physiological processes [15]. It is generally accepted that Hsp90aa1 is the inducible isoform while Hsp90ab1 is constitutively expressed and typically does not respond to stress stimuli [11]. This is the first report to demonstrate that Hsp90ab1 could be induced by an environmental factor such as diet.

Skeletal muscle is a major metabolically active tissue that encompasses vital motor and metabolic functions including post-prandial glucose homeostasis and lipid metabolism. Metabolic plasticity of skeletal muscle is an essential characteristic for maintaining balance of physiological functions based on the availability of substrate within the system during the switch between fed and fasting states to dictate optimized substrate utilization [16]. Impaired skeletal muscle glucose homeostasis and lipid oxidation capacity is associated with insulin resistance, type 2 diabetes, and metabolic disease [17], suggesting the molecular mediators of these pathways may be viable therapeutic targets for metabolic disease and type 2 diabetes. The observed induction in Hsp90ab1 expression in response to a high fat diet in skeletal muscle, but not liver (Additional file 2: Figure S2), of DIO mice, suggests that HSP90ab1 could potentially influence pathways regulating insulin resistance and dysregulation of glucose homeostasis. In the present study, the HSP90ab1 isoform was demonstrated to be a contributor to the pathophysiology of metabolic disease. Knockdown of HPS90ab1 in DIO mice improved glucose tolerance, hyperinsulinemia, and was associated with significant lowering of PDK4 expression. PDK4 is a muscle specific serine kinase that phosphorylates the catalytic subunit of the pyruvate dehydrogenase complex (PDC), PDH E1α, resulting in the inactivation of the complex and preventing the entry of pyruvate generated from glycolysis into mitochondrial Krebs cycle [18]. PDK4 has been reported to act as a cellular homeostat and its activation by the high mitochondrial acetyl-CoA/CoA and NADH/NAD(+) concentration ratios, reflected by high rates of long chain fatty acid (LCFA) oxidation, has been proposed to induce glucose oxidation inhibition by fatty acid oxidation in skeletal muscle [19]. Furthermore, inhibition of PDK4 has been shown to activate the PDC complex and lower elevated blood glucose in insulin resistant animals [20, 21]. Herein, knockdown of HSP90ab1 was associated with a decrease in phosphorylation of three key serine residues in PDH E1α indicative of activation of PDC complex. Consistent with others, these data support that the improved glucose tolerance with Hsp90ab1 knockdown is most likely a consequence of decreased PDK4 expression and concomitant activation of the PDC complex, supporting glucose oxidation. In addition, knockdown of HSP90ab1 significantly reduced fed insulin and glucose and increased skeletal muscle glycogen content, which suggests improvement in skeletal muscle insulin sensitivity and glucose homeostasis in DIO mice.

The data in this study suggests that HSP90ab1 is potentially involved in influencing metabolic plasticity in skeletal muscle by its ability to arbitrate substrate utilization including glucose and fatty acid. In addition to its ability to influence *PDK4* expression and activity as described above, knock-down of HSP90ab1 was associated with alterations in expression several genes regulating fatty acid metabolism including ACADL, ACCA and ATGL, supporting a role in potentially influencing fatty acid utilization. This is further substantiated by the observed increase in skeletal muscle oxygen consumption rate in the presence of palmitate as a metabolic substrate. In the present study knockdown of Hsp90ab1 reduced expression in the skeletal muscle and this was associated with improvement in metabolic flexibility, as systemic administration of ASO to Hsp90ab1 significantly improved impaired glucose tolerance in DIO mice. It must be noted that given the systemic administration of ASO, we cannot exclude the possibility that alterations in other tissues may contribute to the observed findings. In addition, HSP90ab1 may have different functions in other tissues. Future studies are needed to assess whether HSP90ab1 expression in different tissues/organs is affected and/or whether HSP90ab1 may function differently in other tissues/organs.

## Conclusions

The present study demonstrates that Hsp90ab1 is a regulator of skeletal muscle metabolism and suppression of Hsp90ab1 is a valid therapeutic clinically relevant strategy in the management of dysregulated metabolic disease and insulin resistance.

## Additional files

Additional file 1: Figure S1. Knockdown of Hsp90ab1 decreases PDH catalytic subunit phosphorylation. Skeletal muscle of DIO mice were analyzed by Western blot after 4 weeks antisense oligonucleotide (ASO) treatment at 10μg/kg/day. Phosphorylation of PDH E1α was examined at serine 232 (S232) serine 293 (S293), and serine 300 (S300), quantification of Western blot shows that only phosphorylation at S232 was decreased after Hsp90 knockdown (*p < 0.05, *n* = 10 for NC and *n* = 9 for ASO). (PDF 35 kb)

Additional file 2: Figure S2. Hsp90ab1 ASO treatment does not significantly affect protein expression in the liver. Male mice were fed a high fat diet (DIO) for 12 weeks prior to receiving either negative control (NC) ASO or Hsp90ab1 ASO 10μg/kg/day two times a week for 4 weeks. Mice were then sacrificed, liver was collected, and protein expression of Hsp90ab1 was assessed by Western blot. Top panel shows representative Western blot and bottom bar graph represents mean densitometric intensity relative to NC of *n* = 5 NC and n = 10 ASO Hsp90ab1. (PDF 481 kb)

## Abbreviations

ACADL: Acyl-CoA dehydrogenase, long chain
ACCA: Acetyle-CoA carboxylase alpha
ANOVA: Analysis of variance
ASO: Antisense oligonucleotide
ATP: Adenosine triphosphate
AUV: Area under the curve
CCCP: Carbonyl cyanide *m*-chlorophenyl hydrazone
CPT1B: Carnitine palmitoyltransferase 1B
DIO: Diet-induced obesity
ECAR: Extracellular acidification rate (ECAR)
ELISA: Enzyme-linked immunoabsorbent assay
FAO: Fatty acid oxidation
GYS1: Glycogen synthase 1
HSF1: Heat shock factor
HSL: Lipase hormone-sensitive
HSP: Heat shock protein
HSSM: Human skeletal muscle myotubes
i.p.: Intraperitoneal
IPGTT: Intraperitoneal glucose tolerance test
LCFA: Long chain fatty acid
OCR: Oxygen consumption rate
PDC: Pyruvate dehydrogenase complex
PDH: Pyruvate dehydrogenase
PDK4: Pyruvate dehydrogenase kinase isozymes
PFKM: Phosphofructokinase
PNPLA2: Patatin-like phospholipase domain containing 2

## References

[1] De Maio A (1999). Heat shock proteins: facts, thoughts, and dreams. Shock.

[2] Wu C (1995). Heat shock transcription factors: structure and regulation. Annu Rev Cell Dev Biol.

[3] Schlesinger MJ (1990). Heat shock proteins. J Biol Chem.

[4] Becker J, Craig EA (1994). Heat-shock proteins as molecular chaperones. Eur J Biochem.

[5] Lee JH, Gao J, Kosinski PA, Elliman SJ, Hughes TE, Gromada J, Kemp DM (2013). Heat shock protein 90 (HSP90) inhibitors activate the heat shock factor 1 (HSF1) stress response pathway and improve glucose regulation in diabetic mice. Biochem Biophys Res Commun.

[6] Barrott JJ, Haystead TA (2013). Hsp90, an unlikely ally in the war on cancer. FEBS J.

[7] Proia DA, Bates RC (2014). Ganetespib and HSP90: translating preclinical hypotheses into clinical promise. Cancer Res.

[8] Sreedhar AS, Kalmar E, Csermely P, Shen YF (2004). Hsp90 isoforms: functions, expression and clinical importance. FEBS Lett.

[9] Barginear MF, Van Poznak C, Rosen N, Modi S, Hudis CA, Budman DR (2008). The heat shock protein 90 chaperone complex: an evolving therapeutic target. Curr Cancer Drug Targets.

[10] Chae YC, Angelin A, Lisanti S, Kossenkov AV, Speicher KD, Wang H, Powers JF, Tischler AS, Pacak K, Fliedner S (2013). Landscape of the mitochondrial Hsp90 metabolome in tumours. Nat Commun.

[11] Gao Y, Yechikov S, Vazquez AE, Chen D, Nie L (2013). Distinct roles of molecular chaperones HSP90alpha and HSP90beta in the biogenesis of KCNQ4 channels. PLoS One.

[12] Csermely P, Schnaider T, Soti C, Prohaszka Z, Nardai G (1998). The 90-kDa molecular chaperone family: structure, function, and clinical applications. A comprehensive review. Pharmacol Ther.

[13] Zhang S, Hulver MW, McMillan RP, Cline MA, Gilbert ER (2014). The pivotal role of pyruvate dehydrogenase kinases in metabolic flexibility. Nutrition & Metabolism.

[14] Hansen T (2002). Type 2 diabetes mellitus--a multifactorial disease. Ann Univ Mariae Curie Sklodowska Med.

[15] Eckl JM, Richter K (2013). Functions of the Hsp90 chaperone system: lifting client proteins to new heights. Int J Biochem Mol Biol.

[16] Galgani JE, Heilbronn LK, Azuma K, Kelley DE, Albu JB, Pi-Sunyer X, Smith SR, Ravussin E, Look AARG (2008). Metabolic flexibility in response to glucose is not impaired in people with type 2 diabetes after controlling for glucose disposal rate. Diabetes.

[17] Thyfault JP, Rector RS, Noland RC (2006). Metabolic inflexibility in skeletal muscle: a prelude to the cardiometabolic syndrome?. J Cardiometab Syndr.

[18] Kolobova E, Tuganova A, Boulatnikov I, Popov KM (2001). Regulation of pyruvate dehydrogenase activity through phosphorylation at multiple sites. Biochem J.

[19] Holness MJ, Sugden MC (2003). Regulation of pyruvate dehydrogenase complex activity by reversible phosphorylation. Biochem Soc Trans.

[20] Kukimoto-Niino M, Tokmakov A, Terada T, Ohbayashi N, Fujimoto T, Gomi S, Shiromizu I, Kawamoto M, Matsusue T, Shirouzu M (2011). Inhibitor-bound structures of human pyruvate dehydrogenase kinase 4. Acta Crystallogr D Biol Crystallogr.

[21] Roche TE, Hiromasa Y (2007). Pyruvate dehydrogenase kinase regulatory mechanisms and inhibition in treating diabetes, heart ischemia, and cancer. Cell Mol Life Sci.

