# Supplementary figures and images for: Hsp90β knockdown in DIO mice reverses insulin resistance and improves glucose tolerance
Source: Nutr Metab (Lond). 2018 Feb 2;15:11. doi: 10.1186/s12986-018-0242-6 (PMC5796506; doi:10.1186/s12986-018-0242-6)

Figure S1

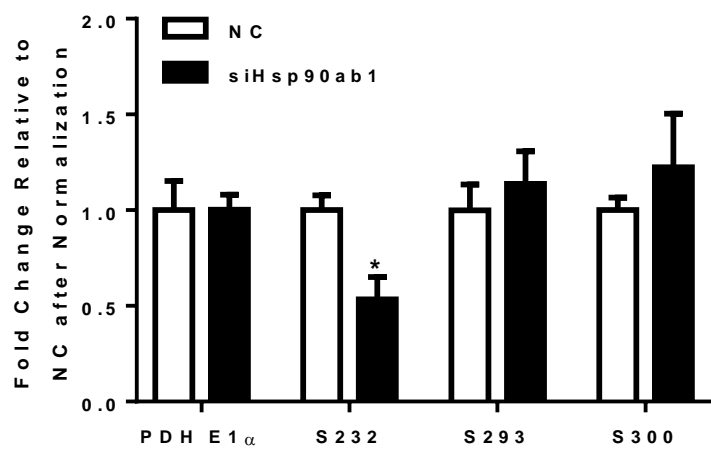

Supplement: Supplementary file 1 — Knockdown of Hsp90ab1 decreases PDH catalytic subunit phosphorylation. Skeletal muscle of DIO mice were analyzed by Western blot after 4 weeks antisense oligonucleotide (ASO) treatment at 10μg/kg/day. Phosphorylation of PDH E1α was examined at serine 232 (S232) serine 293 (S293), and serine 300 (S300), quantification of Western blot shows that only phosphorylation at S232 was decreased after Hsp90 knockdown (*p < 0.05, n = 10 for NC and n = 9 for ASO). (PDF 35 kb) [file 12986_2018_242_MOESM1_ESM.pdf]

Figure S2

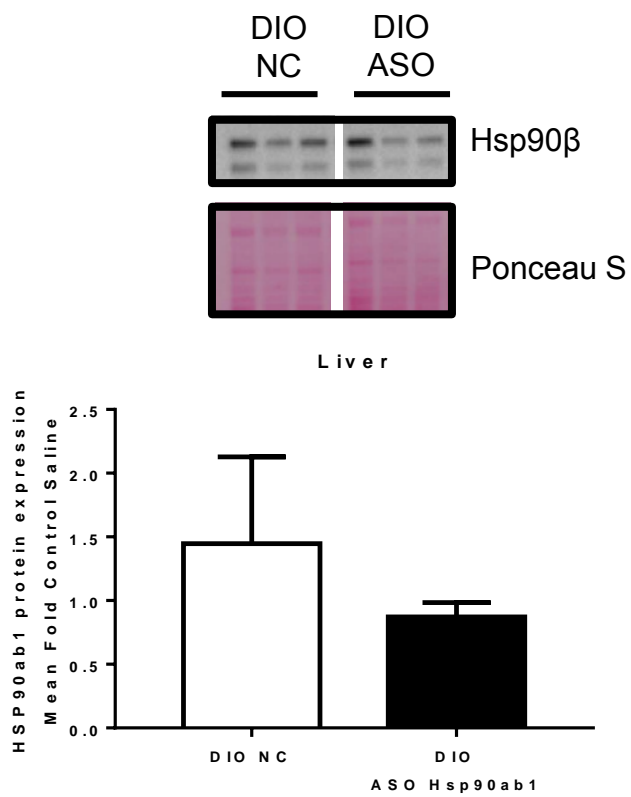

Supplement: Supplementary file 2 — Hsp90ab1 ASO treatment does not significantly affect protein expression in the liver. Male mice were fed a high fat diet (DIO) for 12 weeks prior to receiving either negative control (NC) ASO or Hsp90ab1 ASO 10μg/kg/day two times a week for 4 weeks. Mice were then sacrificed, liver was collected, and protein expression of Hsp90ab1 was assessed by Western blot. Top panel shows representative Western blot and bottom bar graph represents mean densitometric intensity relative to NC of n = 5 NC and n = 10 ASO Hsp90ab1. (PDF 481 kb) [file 12986_2018_242_MOESM2_ESM.pdf]
